# Effects of a Mobile-Based Intervention for Parents of Children With Crying, Sleeping, and Feeding Problems: Randomized Controlled Trial

**DOI:** 10.2196/41804

**Published:** 2023-03-10

**Authors:** Michaela Augustin, Maria Licata-Dandel, Linda D Breeman, Mathias Harrer, Ayten Bilgin, Dieter Wolke, Volker Mall, Margret Ziegler, David Daniel Ebert, Anna Friedmann

**Affiliations:** 1 Social Pediatrics TUM School of Medicine Technical University of Munich Munich Germany; 2 kbo-Kinderzentrum Munich Munich Germany; 3 Health, Medical, and Neuropsychology Unit Leiden University Leiden Netherlands; 4 Psychology & Digital Mental Health Care Department of Sports and Health Sciences Technical University of Munich Munich Germany; 5 Clinical Psychology and Psychotherapy Institute for Psychology Friedrich-Alexander-University Erlangen-Nuremberg Erlangen Germany; 6 School of Psychology University of Kent Canterbury United Kingdom; 7 Division of Health Sciences Warwick Medical School University of Warwick Coventry United Kingdom; 8 Department of Psychology University of Warwick Coventry United Kingdom

**Keywords:** children, crying problems, sleeping problems, feeding problems, feeding, regulatory problems, intervention study, Mobile Health Care, health app, mobile app, patient education, psychoeducation, eHealth, mobile health, mHealth, parenting, baby, babies, sleep, crying, newborn, mobile phone

## Abstract

**Background:**

Excessive crying, sleeping, and feeding problems in early childhood are major stressors that can result in parents feeling socially isolated and having low self-efficacy. Affected children are a risk group for being maltreated and developing emotional and behavioral problems. Thus, the development of an innovative and interactive psychoeducational app for parents of children with crying, sleeping, and feeding problems may provide low-threshold access to scientifically based information and reduce negative outcomes in parents and children.

**Objective:**

We aimed to investigate whether following the use of a newly developed psychoeducational app, the parents of children with crying, sleeping, or feeding problems experienced less parenting stress; gained more knowledge about crying, sleeping, and feeding problems; and perceived themselves as more self-effective and as better socially supported and whether their children’s symptoms decreased more than those of the parents who did not use the app.

**Methods:**

Our clinical sample consisted of 136 parents of children (aged 0-24 months) who contacted a cry baby outpatient clinic in Bavaria (Southern Germany) for an initial consultation. Using a randomized controlled design, families were randomly allocated to either an intervention group (IG; 73/136, 53.7%) or a waitlist control group (WCG; 63/136, 46.3%) during the usual waiting time until consultation. The IG was given a psychoeducational app that included evidence-based information via text and videos, a child behavior diary function, a parent chat forum and experience report, tips on relaxation, an emergency plan, and a regional directory of specialized counseling centers. Outcome variables were assessed using validated questionnaires at baseline test and posttest. Both groups were compared at posttest regarding changes in parenting stress (primary outcome) and secondary outcomes, namely knowledge about crying, sleeping, and feeding problems; perceived self-efficacy; perceived social support; and child symptoms.

**Results:**

The mean individual study duration was 23.41 (SD 10.42) days. The IG reported significantly lower levels of parenting stress (mean 83.18, SD 19.94) after app use compared with the WCG (mean 87.46, SD 16.67; *P*=.03; Cohen *d*=0.23). Furthermore, parents in the IG reported a higher level of knowledge about crying, sleeping, and feeding (mean 62.91, SD 4.30) than those in the WCG (mean 61.15, SD 4.46; *P*<.001; Cohen *d*=0.38). No differences at posttest were found between groups in terms of parental efficacy (*P*=.34; Cohen *d*=0.05), perceived social support (*P*=.66; Cohen *d*=0.04), and child symptoms (*P*=.35; Cohen *d*=0.10).

**Conclusions:**

This study provides initial evidence of the efficacy of a psychoeducational app for parents with child crying, sleeping, and feeding problems. By reducing parental stress and increasing knowledge of children’s symptoms, the app has the potential to serve as an effective secondary preventive measure. Additional large-scale studies are needed to investigate long-term benefits.

**Trial Registration:**

German Clinical Trials Register DRKS00019001; https://drks.de/search/en/trial/DRKS00019001

## Introduction

### Definition

Excessive crying, sleeping, and feeding problems are common sources of stress for parents during early childhood [1,2]. They are characterized by self-regulation difficulties [2], which may manifest as fussiness, prolonged crying, difficulties transitioning to sleep or maintaining sleep, or refusal of new or selective foods [3]. Some children may experience a single problem, whereas others experience multiple problems simultaneously or persistently from early childhood to toddlerhood [2,4].

### Prevalence

The prevalence rates of crying, sleeping, and feeding problems vary widely depending on the exact definition of the problems, diagnostic procedures, and the study population. For excessive crying, the incidence rates range from 5% to 26% in the first 18 months of life [5-7]. Sleeping problems in early childhood are evident in 10% to 33.3% of children aged <24 months [7-11], whereas mild to moderate feeding difficulties occur in 20% to 43% of children in the first year of life [7,10,12,13]. Two or more simultaneous problems of feeding, sleeping, or excessive crying emerge in approximately 1% to 26.6% of children in their first 2 years of life [7,10,14]. Although in many cases early crying, sleeping, and feeding problems are transient [14-16], persistent problems are associated with short- and long-term negative consequences for child health and development.

### Associated Short- and Long-term Risks

Several factors are associated with the development of excessive crying, sleeping, and feeding problems, and they include neurodevelopmental vulnerability, high levels of parenting stress, parental pre- and postnatal psychopathology such as depression and anxiety, and impaired parent-child relationships [10,14,17-20]. In addition to enormous exhaustion and dejection, parents often feel helpless and incompetent in their parenting skills, which can be reinforced by persistent crying despite all their efforts to calm and satisfy their babies [2,21-23]. Furthermore, affected families often report that they feel increasingly socially isolated and lack support from their social environment [2,24,25]. Affected families bear a high risk of stressful experiences in which childcare demands outbalance parental resources. Thus, children who cry excessively are considered a specific risk group for child maltreatment, for example, by shaking the child, which can have severe long-term and even life-threatening consequences (shaken baby syndrome) [26-29]. Impairment of the parent-child relationship may persist for several years [14,30-32]. Other long-term consequences include the persistence of sleeping and feeding problems beyond the first months of life up to preschool and even elementary school age [15,16,33] and an increased risk of developing other mental health problems, such as emotional and behavioral problems, as well as cognitive deficits [1,4,33-37]. There is emerging evidence that multiple or persistent crying, sleeping, and feeding problems may have long-lasting effects on attention and avoidant personality symptoms in adulthood [4,27,38]. In summary, early crying, sleeping, and feeding problems are associated with high family stress and increase the risk of impaired child development.

### Psychoeducation as a Suitable Intervention Tool for Affected Families

Thus, it is necessary to provide support to affected families as early as possible to reduce parenting stress. This can contribute to preventing crisis situations as well as child and parent mental health problems in the short and long term [39-41]. Psychoeducation is generally an essential tool for the prevention and treatment of mental illness and stress. In addition to increasing knowledge about the child’s problem or disorder [42,43], psychoeducational interventions can promote empowerment by improving subjective coping strategies [44-46]. This is also relevant for families of children with early crying, sleeping, and feeding problems, as parents consider corresponding reliable information helpful for understanding children’s symptom patterns and signals [24,47,48]. Moreover, Gilkerson et al [49] found that an early psychoeducational intervention program for parents of excessively crying children improved parenting self-efficacy. Findings suggest that psychoeducational interventions not only have a positive impact on parental parameters but can also reduce early child symptoms such as sleeping problems [50]. Furthermore, Hiscock et al [47] demonstrated the positive effects of a psychoeducational intervention on sleeping and crying problems in a subgroup of children who were fed very frequently.

### Need for a Low-Threshold Intervention

Although early intervention is necessary and helpful for affected families, there could be barriers to seeking professional counseling. For example, parents might fear that they will be judged negatively by their social environment as well as by professional health care workers [24]. One possible way to reach affected families at an early stage is by a smartphone app. In Germany, approximately 87% of people have smartphones in their households [51,52]. With >101,000 health- and fitness-related offers in app stores worldwide in 2020 [53], apps are an increasingly popular way to obtain health-related information. Research indicates positive impacts of psychoeducational apps on both parent and child outcomes. Shorey et al [54] showed that an app-based educational program increases parental self-efficacy, social support, and parenting satisfaction during the postpartum period. App-based sleep interventions were found to reduce sleeping problems in children and improve sleep patterns in children aged 6 to 12 months [55] and from the age of 2 years [56]. Furthermore, parents who used features such as tracking feeding behavior reported higher perception of control and self-efficacy [57]. These results indicate that digitized psychoeducational interventions could be effective tools to support families with children with early crying, sleeping, and feeding problems. However, most apps provided in app stores are neither tested for effectiveness nor based on scientifically sound content [58]. Furthermore, most apps such as specialized sleeping or feeding apps target only one of the symptoms [55,56], although the symptoms frequently occur simultaneously in a complex manner [2,4,14]. To our knowledge, no apps are available to date that specifically target crying, sleeping, and feeding problems as common symptom patterns in early childhood. In addition, many apps (such as mere tracking apps) are limited to a few functions, instead of combining psychoeducational and interactive tools [59], and rarely address both child symptoms and parental outcomes, for example, parenting stress. Thus, we developed a new psychoeducational app targeting both parental and child outcomes as a low-threshold early support offer for affected families. In addition to scientifically based information on crying, sleeping, and feeding problems, the app included interactive tools such as a symptom diary function, relaxation strategies for parents, an emergency plan, and contact information for professional counseling centers.

### Study Aim

In this study, we aimed to evaluate the effectiveness of the app in a clinical sample by including an intervention group (IG) and a waitlist control group (WCG). We hypothesized that, compared with the WCG, the IG using the app would have a significant reduction in the primary outcome, that is, parenting stress. Furthermore, we aimed to explore the app’s effects on secondary outcomes, including parental knowledge about crying, sleeping, and feeding problems; parental self-efficacy; perceived social support; and child crying, sleeping, and feeding problems.

## Methods

### Study Design

The app was evaluated in a monocentric, prospective, and randomized controlled intervention study using a pretest-posttest (posttest [t2]) design and a WCG from 2019 to 2022. The methods and results of this study are presented in accordance with the CONSORT (Consolidated Standards of Reporting Trials) Statement [60], the CONSORT-EHEALTH (CONSORT of Electronic and Mobile Health Applications and Online Tele Health) checklist (Multimedia Appendix 1), and the Guidelines for Executing and Reporting Research on Internet Interventions [61].

### Ethics Approval

The study protocol was approved by the Ethics Committee of the Technical University of Munich (vote number: 56/18 S). The study was registered with the German Register of Clinical Studies (DRKS; register number: DRKS00019001; Multimedia Appendix 2).

### Recruitment and Procedure

The target group were German-speaking parents of children aged 0 to 24 months who contacted a cry baby outpatient clinic in Bavaria (Southern Germany) for the first consultation because of crying, sleeping, or feeding problems. To avoid confounding the effects of app use with the effects of counseling, the individual study phase ended before the first appointment at the outpatient clinic (posttest [t2]). Participants whose possible study duration was very short (<10 days from first contact with the study team until counseling appointment in the outpatient clinic) were excluded because the applied questionnaires referred to a report period of at least 1 week. During the initial phone contact with the clinic, interested parents who met the inclusion criteria were referred to the study team. After verbal consent, study information, declaration of consent, and baseline test questionnaires (t1) were sent to their home addresses. As soon as the study team received the original signed informed consent and t1 questionnaires, families were included and randomly assigned to the IG or the WCG. Randomization was conducted by an independent researcher using Research Randomizer [62]. Participants were not blinded to the study conditions, and they received access to the app by email at different time points. While the IG obtained the app for the duration of the regular waiting period until the first counseling appointment, the WCG received it only after the first counseling appointment. A few days before the counseling appointment, t2 questionnaires were sent by post to the participants, and completed forms were collected by the study team just before the counseling appointment. To avoid sequence effects, all questionnaires were administered in permuted order. The individual study duration corresponded to the average waiting time for an initial counseling appointment (mean 3 weeks). During the study, participants were contacted repeatedly via email to receive a confirmation or reminder: (1) after 1 week if they had not returned the t1 questionnaires by then; (2) after the t1 questionnaires arrived; (3) five days after the app was unlocked to see whether the installation was successful; (4) one week before the initial counseling appointment in the outpatient clinic to remind them to bring the completed t2 questionnaire; and (5) after the study ended with a brief thank you note for participation.

### Intervention

**Figure 1:**
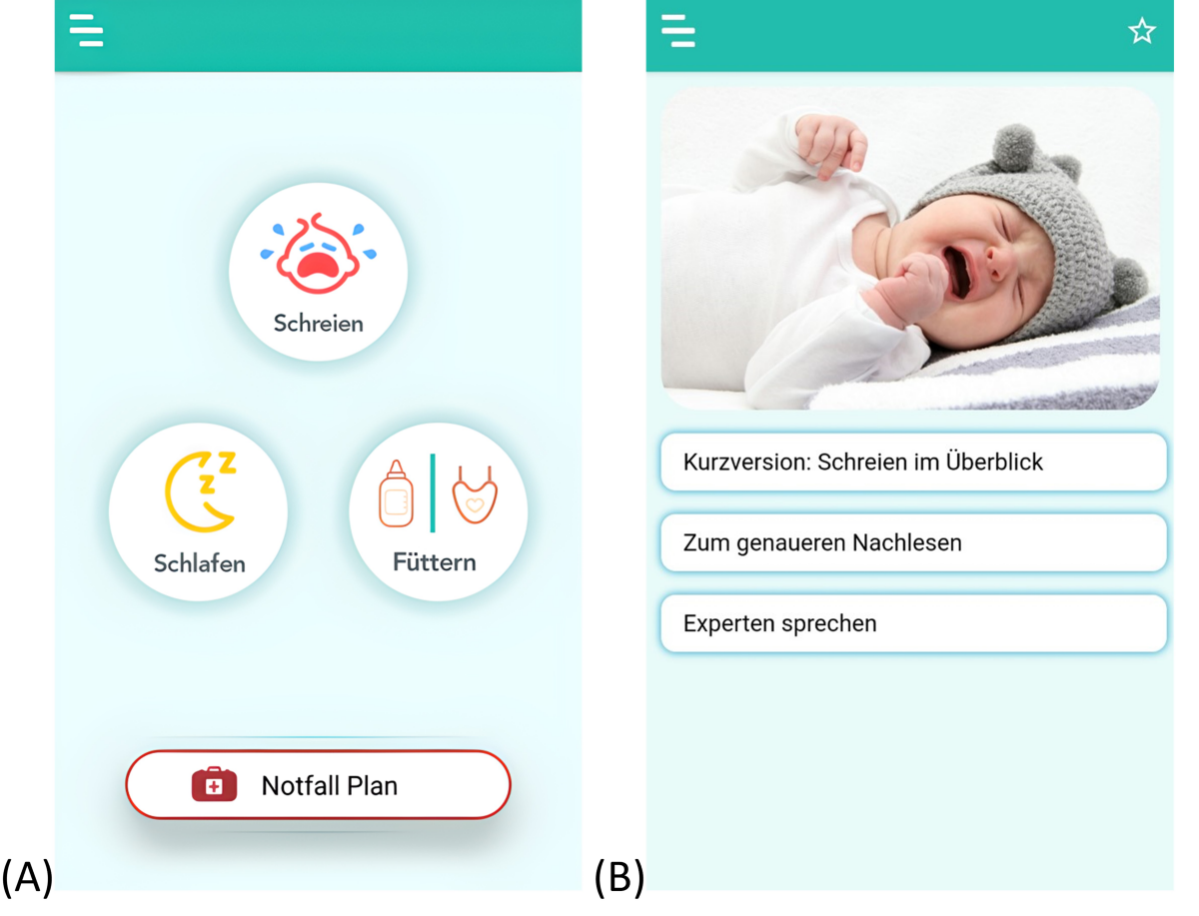
App intervention: screenshot examples. (A) Main screen and (B) menu for psychoeducational content concerning child crying.

The overall aim of the app was to provide psychoeducation regarding early childhood crying, sleeping, and feeding problems (for screenshot examples, see Figure 1). In line with the Medical Research Council guidelines [63], the app was developed in a working consortium of international scientists and clinical health care professionals (n=10) in the field of early crying, sleeping, and feeding problems and was piloted regarding its first impression by parents and clinical experts (n=15; M Augustin et al, unpublished data, December 2022). The first final version of the app consisted of the following main components: (1) informational texts including tips on how to deal adequately with a child’s behavior were provided in a short version in simple language as well as in a more detailed version; (2) video interviews with experienced clinical experts addressing frequent questions; (3) an emergency plan in acute situations of excessive demands providing guidance for de-escalation; (4) a profile and diary function enabling parents to document their child’s symptoms; (5) self-care strategies suitable for everyday use addressing parents’ own needs; (6) an experience report of one family with a child with crying problems showing frequent problems of affected families; (7) a chat forum providing the opportunity to contact other affected parents; and (8) a regional register of counseling centers in Bavaria aiming to encourage affected parents to seek professional support at an early stage. Parents had the app at their free disposal and could decide how often they wanted to use it.

### Measures

#### Primary Outcome: Parenting Stress

Parenting stress was assessed at t1 and t2 with the Eltern-Belastungs-Inventar (EBI) [64], which is a German adaptation of the Parenting Stress Index [65]*.* The questionnaire contained 48 items covering the child domain (stress emanating from the child’s behavior) and the parent domain (impairments of parental functions). In this study, the overall parent domain score was applied as the outcome variable, and the 7 parent domain subscales (attachment, isolation, parental competence, depression, health, role restriction, and spouse-related stress) were used in the secondary exploratory analysis. Responses were given on a 5-point-Likert scale (1=strongly agree to 5=strongly disagree). The internal consistency (Cronbach *α*) of the EBI parent domain has been shown to be excellent (.93). External validity has been examined in validation studies using different samples. Results showed, among others, moderate to high correlations of the EBI with other stress indicators, as well as related constructs of parenting stress [66].

#### Secondary Outcomes

##### Knowledge About Crying, Sleeping, and Feeding

A multiple-choice test on crying, sleeping, and feeding (self-developed and based on the contents of the app) assessed the parents’ level of knowledge at t1 and t2 using 18 questions with 4 answer options, of which one or more were correct. An example item is as follows: “It is natural for children to cry (eg, due to tummy ache). However, at a certain age, the crying frequency decreases in most babies. When does this occur?—after 6 weeks—from 3 months of life—from 6 months of life—from 12 months of life.” The maximum possible score was 72. The internal reliability was acceptable, with a Cronbach *α* of .72.

In a face validity test, participants rated all items as very suitable or mostly suitable (Table S1 in Multimedia Appendix 3).

##### Parental Self-efficacy

The Perceived Maternal Parenting Self-Efficacy Questionnaire (PMP-SE) [67] measured parental self-efficacy in dealing with the child at t1 and t2. A total score was computed based on 20 items with 4 subscales (caretaking procedures, evoking behaviors, reading behaviors or signaling, and situational beliefs). Items were scored on a 4-point Likert scale ranging from 1 (do not agree at all) to 4 (totally agree). Internal reliability has been proven to be excellent, with a Cronbach *α* of .91. The predictive and criterion validity have been previously reported [67].

##### Perceived Social Support

Perceived social support was assessed at t1 and t2 with the K-22 short form of the Social Support Questionnaire (F-SozU) [68], and the total score was computed based on 22 items with 3 subscales (practical support, emotional support, and social integration). All items were rated on a 5-point Likert scale (1=does not apply to 5=is exactly correct). Internal reliability of the total score has been proven to be excellent with a Cronbach *α* of .91 [69]. Its validity has been confirmed in numerous studies [68,69].

##### Child Crying, Sleeping, and Feeding Problems

The Questionnaire for Crying, Feeding, and Sleeping (CFS; German Version) [70] assessed early child problems with respect to crying, sleeping, and feeding at t1 and t2. A total score was computed based on 3 scales: “Crying, Whining, and Sleeping” (24 items), “feeding” (13 items), and “coregulation” (12 items). Cronbach *α* has been shown to vary from .81 to .89 for the subscales, and *α* is .90 for the complete questionnaire, indicating high internal reliability. The questionnaire has been validated by correlations with behavioral diaries and clinical diagnoses [70].

##### Sociodemographic Questionnaire (Self-developed)

Parental age, participant’s relation to child, nationality, mother tongue, educational qualifications, current employment situation, partnership status, child’s age and gender, siblings, and information about the child’s problems were recorded at t1.

##### App Use

App use was measured at t2 with 1 item derived from an app evaluation questionnaire (self-developed) using 5 categories: daily (4), several times a week (3), once a week (2), <once a week (1), and app not used (0).

### Statistical Analysis

#### Power

An a priori case number calculation was performed using G*Power software (version 3.1.9.2; Kiel University) [71]. A repeated-measures mixed ANOVA (between-group × within-subject factor) was assumed. However, to exclude between-subject effects with sufficient certainty, case number planning was completed for the between-subject factor analyses, providing lower power. The estimation was based on a type 1 error of *α*=.01 and a power of 1–*β*=.80. Findings from a randomized controlled trial (RCT) of the effectiveness of a psychoeducational app for parents in the postpartum period [54] and another RCT on the effectiveness of an information video–based intervention for early child crying [72] were used to estimate effect sizes [54]. On the basis of statistical values reported in these studies, calculations yielded medium to high effect sizes. Accordingly, in this study, the number of cases was estimated more conservatively for medium treatment effects (Cohen *f*=0.30), using the ANOVA design described previously. The calculation of the number of cases resulted in a sample size of 136 participants.

#### Analysis Plan

To evaluate the effectiveness of the app-based intervention in the IG compared with the WCG, analyses were based on the intention-to-treat (ITT) principle. All analyses were performed using R software (version 4.0.1; R Foundation for Statistical Computing) [73]. The code used for the analyses has been made openly available in an Open Science Framework repository [74]. Missing values were assumed to be missing at random, which is typically plausible for trials with off-treatment assessments [75,76]. Missing values were therefore imputed using groupwise multivariate imputation by chained equations algorithm (fully conditional specification) [77], with 50 iterations and 50 (m) imputation sets. Several auxiliary variables were included in the imputation model to approximate the missing data in random missingness patterns.

#### Main Effectiveness Analysis

We tested whether the outcomes of app-based intervention in the IG was superior to those of the WCG in terms of its effects on parenting stress and secondary outcomes from pre- to posttests. For the confirmatory primary outcome analysis, a 1-sided test was used, whereas 2-sided testing was used for exploratory secondary outcome analyses. As an additional exploratory analysis, we examined the differences between groups on the subscales of the primary outcome (EBI parental stress). The effect differences between the 2 study conditions were assessed using univariate analysis of covariance. Baseline scores were used as covariates. Child age was entered as an additional covariate for the child symptom outcome analysis (CFS). All the models were fitted to each of the multiple imputed data sets. Model estimates were then aggregated via Rubin’s combination rules [78] using a large-sample *χ*^2^-approximation to combine the *F*-statistics [79]. To calculate the between-group standardized mean difference (Cohen *d*), we pooled the unstandardized group coefficients of a linear model without covariate adjustment using Rubin’s rules. This estimate was then standardized using the pooled outcome SD to obtain Cohen *d* and CI.

#### Sensitivity Analysis

To examine the robustness of the main analysis, 2 sensitivity analyses were conducted. First, we conducted a completer analysis of individuals who had no missing data and provided data at all assessment points. Second, a per-protocol analysis was conducted, focusing on individuals in the IG who accessed the app at least once (while retaining all participants in the WCG). The applied methods exactly mirrored the ones of the main effectiveness evaluation.

## Results

### Participant Enrollment and Characteristics

**Figure 2:**
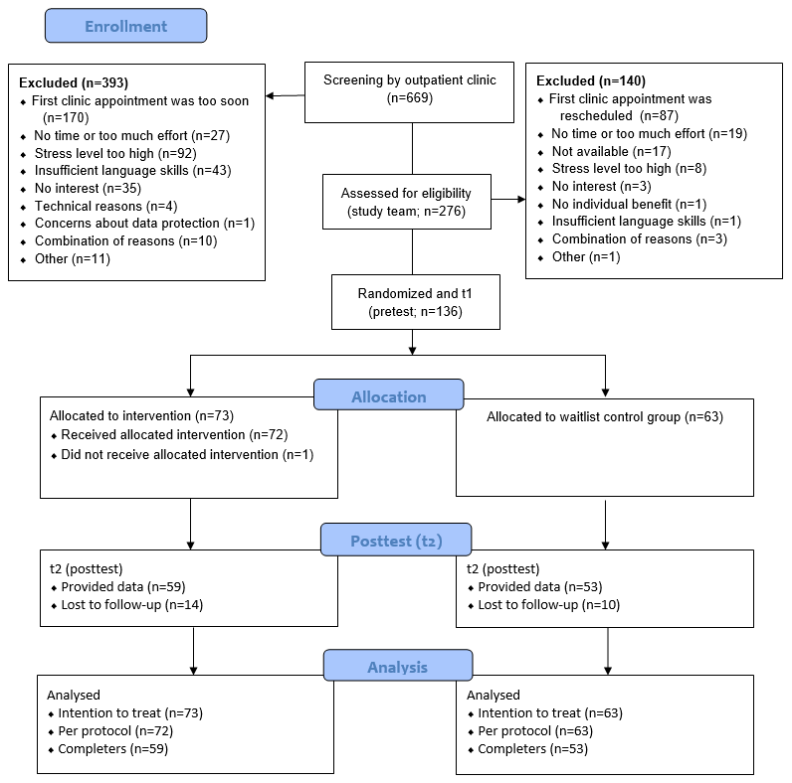
Participant flowchart.

After the first screening by the outpatient clinic, a total of 41.3% (276/669) of participants were assessed for eligibility by the study team. A total 136 individuals who met the inclusion criteria were randomly allocated to the IG (n=73, 53.7%) and WCG (n=63, 46.3%; Figure 2 shows the CONSORT participant flowchart). Participants (mean age 33.97, SD 4.03 years) were predominantly mothers (127/136, 93.3%) of German nationality (113/136, 83.1%) with higher education (105/136, 77.2% qualified for university entrance) and currently not employed or on parental leave (97/136, 71.3%). Children (70/136, 51.5% boys) aged on average 10.32 (SD 5.06) months, had no siblings (95/136, 69.9%), and experienced sleeping (61/136, 44.9%) or combined (40/136, 29.4%) problems. The demographics divided by group are presented in Table 1.

**Table 1:** Participant characteristics.

Characteristics		Intervention group (n=73)	Waitlist control group (n=63)
Parental age (years), mean (SD)		33.71 (3.86)	34.27 (4.22)
**Participant’s relation to child, n (%)**			
	Mother	68 (93.2)	59 (93.7)
	Father	5 (6.8)	4 (6.3)
**Academic qualification, n (%)**			
	Qualified for university entrance	57 (78.1)	48 (76.2)
	Other or missing	16 (21.9)	15 (23.8)
**Nationality, n (%)**			
	German	63 (86.3)	50 (79.4)
	Other	10 (13.7)	13 (20.6)
**First language, n (%)**			
	German	58 (79.5)	52 (82.5)
	Other	15 (20.5)	11 (17.5)
**Employment, n (%)**			
	Parental leave or currently not employed	53 (72.6)	44 (69.8)
	Currently employed or apprenticeship	19 (26.0)	17 (27)
	Other or missing	1 (1.4)	1 (1.6)
Single parent, n (%)		0 (0)	2 (3.2)
Child age (months), mean (SD)		10.21 (4.95)	10.44 (5.21)
**Child gender, n (%)**			
	Girl	36 (49.3)	30 (47.6)
	Boy	37 (50.7)	33 (52.4)
**Siblings, n (%)**			
	Yes	22 (30.1)	19 (30.2)
	No	51 (69.9)	44 (69.8)
Child’s symptom duration (months), mean (SD)		6.91 (4.20)	7.76 (5.46)
**Reason for consultation, n (%)**			
	Sleeping problems	34 (46.6)	27 (42.9)
	Feeding problems	9 (12.3)	7 (11.1)
	Crying or whining	5 (6.8)	3 (4.8)
	Combined problems	21 (28.8)	19 (30.2)
	Other or missing	4 (5.5)	7 (11.1)

### Descriptive Statistics

Mean study duration from pre- to posttest was 23.41 (SD 10.42; range 10-49) days. In the IG, most parents used the app at least once a week (once a week: 24/73, 33%; several times a week: 21/73, 29%; daily: 7/73, 10%), whereas 27% (20/73) used the app less than once a week and 1 person did not use the app for unknown reasons. Descriptive statistics of outcome variables are displayed in Table 2.

**Table 2:** Mean scores in intention-to-treat sample per study group for outcome variables (pretest and posttest).

Outcome variable			Descriptive statistics of outcomes						
			Intervention group, mean (SD)				Waitlist control group, mean (SD)		
			t1 (pretest)		t2 (posttest)		t1 (pretest)		t2 (posttest)
**EBI^a^ parental scale**			85.31 (18.97)		83.18 (19.94)		86.39 (16.65)		87.46 (16.67)
	Attachment	10.05 (3.16)		10.35 (3.24)		9.86 (3.28)		10.21 (3.26)	
	Isolation	10.86 (4.04)		10.57 (3.86)		11.11 (3.38)		11.81 (3.51)	
	Parental competence	11.14 (3.94)		10.80 (3.91)		11.27 (3.76)		11.10 (3.41)	
	Depression	13.14 (4.00)		12.32 (4.01)		12.96 (3.06)		12.22 (3.26)	
	Health	13.10 (3.28)		12.23 (3.33)		13.19 (3.59)		13.02 (3.58)	
	Role restriction	13.15 (4.31)		13.27 (4.08)		13.48 (3.51)		14.13 (3.27)	
	Spouse-related stress	13.88 (3.51)		13.65 (3.90)		14.52 (3.10)		14.97 (3.11)	
Knowledge test			59.39 (4.60)		62.91 (4.30)		60.66 (5.04)		61.15 (4.46)
PMP-SE^b^			65.38 (6.78)		66.96 (7.15)		65.89 (6.74)		66.62 (6.32)
F-SozU^c^			4.35 (0.58)		4.35 (0.60)		4.18 (0.68)		4.17 (0.68)
CFS^d^			2.28 (0.27)		2.25 (0.28)		2.29 (0.23)		2.22 (0.25)

^a^EBI: Eltern-Belastungs-Inventar.

^b^PMP-SE: Perceived Maternal Parenting Self-Efficacy Questionnaire.

^c^F-SozU: Social Support Questionnaire.

^d^CFS: Questionnaire for Crying, Feeding, and Sleeping.

### Main Effectiveness Evaluation

#### Primary Outcome

Participants in the IG reported significantly lower levels of parenting stress at t2 compared with participants in the WCG (*F*_1,2683.50_=3.38; *P*=.03; Cohen *d*=−0.23). Exploratory analysis of EBI parent domain subscales revealed a significant reduction in the social isolation subscale (*F*_1,1072.48_=4.68; *P*=.03; Cohen *d*=−0.32), but not in the other domains (Table 3).

**Table 3:** Analysis of covariance (ANCOVA) results for intention-to-treat sample.

Variable			*F* test (*df*^a^)		*P* value		Cohen *d*		95% CI
**EBI^b^ parental subscale**			3.38 (1,2683.50)		.03^c^		−0.23		−0.52 to −0.06
	Attachment	0.10 (1,12563.63)		.76		0.04		−0.30 to 0.38	
	Isolation	4.68 (1,1072.48)		.03^d^		−0.32		−0.66 to 0.02	
	Parental competence	0.25 (1,4046.88)		.62		−0.08		−0.42 to 0.26	
	Depression	0.11 (1,6522.79)		.74		0.03		−0.31 to 0.37	
	Health	2.24 (1,1536.52)		.13		−0.22		−0.56 to 0.12	
	Role restriction	2.31 (1,5263.57)		.13		−0.22		−0.57 to 0.11	
	Spouse-related stress	3.17 (1,1136.55)		.08		−0.36		−0.71 to −0.02	
Knowledge test			19.46 (1,402.98)		<.001^d^		0.38		0.05 to 0.73
PMP-SE^e^			0.93 (1,7680.23)		.34		0.05		−0.29 to 0.39
F-SozU^f^			0.20 (1,11479.08)		.66		0.04		−0.10 to 0.17
CFS^g^			0.86 (1581.14)		.35		0.10		−0.23 to 0.45

^a^Approximated *df* based on multivariate imputation.

^b^EBI: Eltern-Belastungs-Inventar.

^c^1-tailed test based on the *t* distribution.

^d^2-tailed test (*F* test).

^e^PMP-SE: Perceived Maternal Parenting Self-Efficacy Questionnaire.

^f^F-SozU: Social Support Questionnaire.

^g^CFS: Questionnaire for Crying, Feeding, and Sleeping.

#### Secondary Outcomes

In the IG, a significantly higher level of knowledge about crying, sleeping, and feeding was noticeable at t2 compared with the WCG (*F*_1,402.98_=19.46; *P*<.001; Cohen *d*=0.38).

No differences were found in parental efficacy, perceived social support, and child symptoms (Table 3).

### Sensitivity Analysis

In the completer analysis, the primary and secondary outcomes remained stable. In line with the ITT analyses, in the IG, significantly lower levels of parenting stress (*P*=.01) and a significantly higher level of knowledge about crying, sleeping, and feeding (*P*<.001), but not in the other domains, were noticeable at t2 compared with the WCG. Furthermore, in the exploratory completer analysis referring to EBI parental subscales, in line with the ITT analysis, significantly lower scores on the social isolation subscale were noticeable (*P*=.004), and, in contrast to the ITT analysis, significantly lower levels on the role restriction (*P*=.04) and spouse-related stress (*P*=.01) subscales were evident in the IG at t2 (Table S2 in Multimedia Appendix 3).

In the per-protocol analysis, one person in the IG who did not use the app was excluded. In line with the ITT and completer analyses, in the IG, significantly lower levels of parenting stress (*P*=.04) and a significantly higher level of knowledge about crying, sleeping, and feeding (*P*<.001), but not in the other domains, were evident at t2 compared with the WCG. In the exploratory analysis of the EBI subscales, a significantly lower score on the spouse-related stress subscale became evident (*P*=.04), which is contrary to the ITT analysis but consistent with the completer analysis (Table S3 in Multimedia Appendix 3).

## Discussion

### Principal Findings

To the best of our knowledge, this is the first randomized controlled clinical study to target an app-based intervention for families having children with crying, sleeping, and feeding problems as common symptom patterns in early childhood. We found significantly lower levels of parenting stress after app use in the IG compared with the WCG. Furthermore, parents in the IG reported a higher level of knowledge about crying, sleeping, and feeding than the parents in the WCG. However, there were no significant differences between the 2 groups in terms of parental efficacy; perceived social support; and child crying, sleeping, and feeding symptoms.

Consistent with other research on families of children with crying, sleeping, and feeding problems [18,80,81], initial parenting stress levels in our sample were very high compared with normative values (EBI parental subscale scores >85 were above the 98 percentile and above the cutoff for individuals with very high stress levels). The finding that the use of our psychoeducational app contributed to the reduction in parenting stress is in line with several previous studies investigating the effects of psychoeducational interventions on stress [82,83]. Psychoeducation can bring various positive effects, such as emotional relief and promotion of coping strategies, beyond a mere increase in knowledge [84,85]. In addition, research shows that parents of children aged <5 years very frequently read web-based information on child health but tend to miss the accuracy of the content they find, are only moderately satisfied with its reliability, and often feel the need to cross-check information [86], which might be potentially time-consuming and tedious. By providing a collection of evidence-based and reliable information about their child’s (problem) behavior as well as intervention strategies, the app use could potentially have spared parents’ time and effort. Further research would need to be sought in this regard.

Exploratory analysis revealed a significant reduction in the parental stress (EBI) subscale of social isolation, and in the sensitivity analysis, effects on the EBI subscales of role restriction and spouse-related stress became evident. Regarding social isolation, this finding is in line with Shorey et al [54], confirming the effects of psychoeducational interventions on perceived support from the social environment. Regarding role restriction, some studies addressing different target groups indicate positive intervention effects (eg, effects of a web-based mindful parenting training for parents of toddlers [87]); however, there is a lack of comparative studies targeting role restriction in the context of child crying, sleeping, or feeding problems. Addressing spouse-related stress, findings indicate that psychoeducational interventions in the pre- and postpartum periods, including both partners, have a positive impact on partnership quality [88,89]. However, we have no information on whether in our sample both partners used the app together, so the result of reduced spouse-related stress in the context of app use still needs further investigation.

With regard to the secondary outcomes, app use led to an increase in knowledge about crying, sleeping, and feeding problems. As summarized in a systematic review by McDowall et al [43], several studies have shown increased knowledge about children’s sleep after educational interventions. For instance, a pilot study by Jones et al [42] revealed that a brief brochure–based psychoeducational intervention enhanced parental knowledge about their children’s sleep in a clinical sample. Although the mechanisms of psychoeducation lack systematic research [90], these findings are in line with the “Information Models” of psychoeducation, which emphasizes the importance of providing families with knowledge about psychological symptoms and their management to create awareness and to contribute to the management of the problems [85].

In this study sample, no effects of app use on parental perceived self-efficacy, social support, or children’s symptoms were evident. With regard to self-efficacy, the absence of effects contrasts with the findings of Gilkerson et al [49] but is in line with the findings of a study by Missler et al [91] who also found no effects of a low-intensity psychoeducational program on parental self-efficacy. In terms of perceived social support, our findings differ from those of other studies focusing on apps for the postpartum period: Shorey et al [54] found that the use of a psychoeducational app increased perceived social support. Regarding child symptoms, studies investigating the effectiveness of psychoeducational interventions on the symptoms of excessive crying, sleeping, and feeding problems have shown inconsistent results. Although some studies indicate that educational interventions can reduce sleeping and crying problems in children [50,55], other studies cannot confirm these effects. In line with our results, Missler et al [91] found no effects of a low-intensity psychoeducational program on perceived problems of child crying, sleeping, and feeding. Hiscock et al [47] found effects only for specific subgroups, namely, very frequently fed children, showing changes in daytime sleep but not in nighttime sleep problems.

One explanation for the missing effects of app use on parental self-efficacy and perceived social support could be that, surprisingly and in contrast with other studies reporting impairment in self-efficacy and social support [24,25,92-95], the mean values of both these outcomes were in the normal range when compared with those of validation studies and normative values [67,96], indicating that these were not major issues in our clinical sample. This difference could be attributed to the characteristics of our sample, which included predominantly highly educated mothers in partnerships. Studies indicate that higher socioeconomic status is associated with higher self-efficacy and social support [97-99]. In addition, sleeping problems were predominant in our sample. Although associations between perceived social support and child crying as well as feeding problems have been investigated in various studies [24,25], studies addressing child sleeping problems are scarce. However, the effects of app use became evident in the EBI social isolation subscale, which might give us an indication that the app could have a positive effect on perceived social isolation. Further research would be useful at this point.

Another possible explanation for the absence of the effects on parental self-efficacy, perceived social support, and child problems could be that these constructs may not be as susceptible to short-term influence as parenting stress or knowledge. A meta-analysis by Amin et al [100] found that educational interventions for parents lasting at least 10 weeks produced significantly greater effects on parental self-efficacy than shorter interventions. To date, there are no comparative data on the extent to which the length of the intervention affects perceived social support. Regarding child symptom reduction, child problem behavior might be moderated by parenting stress; a regularly associated factor is poor parenting behavior, which in turn is linked to poorer child development and more behavioral problems [101,102]. Hence, an effect on child symptoms could become evident after the transfer of learned functional behavioral strategies in everyday life. A longer app use duration could have yielded different results; however, implementation was not possible in this study because the participation of the clinical sample was linked to the waiting time for an initial consultation appointment at the outpatient clinic.

Furthermore, the absence of effects could be anchored in the relevance of child problems to parental outcomes. Self-efficacy theory states that the success of strategies and actions is a major factor in increasing self-efficacy [103]. In the context of crying, sleeping, and feeding problems, this means that child symptoms would need to change because of parental strategies. Because CFS scores indicated clinically relevant problems in our sample and no changes in child symptoms were evident in this study, the effects on self-efficacy may have been absent. The lack of effect on child symptom reduction could also be related to the absence of effect on perceived social support in our study. Owing to the persistence of the child symptoms, parental networking behavior may not have changed.

Finally, we must acknowledge the possibility that an app-based psychoeducational intervention for this clientele is not sufficient to influence child symptoms, self-efficacy, or perceived social support. Parental feelings of helplessness, incompetence, and uncertainty about how to act are crucial therapeutic themes in affected families [2,21-23]. Working on these topics may require more intensive and individualized support such as parent-child psychotherapy or specialized counseling.

Our findings are of specific clinical relevance for the following reasons. First, increased knowledge may, to some extent, lead to altered and more favorable parenting behavior and thus might promote more adequate handling of the child in challenging situations [39-41,85]. Second, stress reduction provides the basis for breaking the vicious cycle of dysfunctional parent-child interactions, which is in turn an important protective factor for child mental health in the context of regulatory skills [2,40,41]. Reducing parenting stress is a prerequisite for increasing parental sensitivity, including the ability to adequately focus on a child’s needs and signals [104]. Third, regarding the long-term consequences of early crying, sleeping, and feeding problems, there is evidence that parenting stress partially mediates the association between early behavioral problems and later mental health problems [102]. Thus, stress reduction might be an important factor in the prevention of long-term effects on healthy child development.

The effect sizes on parenting stress are considered small in this study. Other studies investigating the effects of psychoeducational interventions on stress have also found small effect sizes [83]. Nevertheless, our finding that an effect was evident even after a short application period is promising in terms of app use as a secondary preventive service. From a health economic perspective, it should be pointed out that an app, which is relatively cost-efficient, can reach many people in the community; thus, even small effect sizes can be highly effective at the population level. In terms of knowledge gain, the small effect size might be related to sample characteristics, and research indicates that knowledge about child sleeping problems is positively associated with parental educational level in a clinical sample [105]. In our highly educated sample, it is conceivable that the parental level of knowledge about crying, sleeping, and feeding was already high at baseline, and therefore, only a small effect on knowledge level was obtained. However, we did not compare the different educational levels in our study. Therefore, future research should be conducted in this regard. Furthermore, our sample had been dealing with the child’s symptoms for an average of 7 months before seeking professional help in the outpatient clinic. Research shows that parents of children tend to seek internet-based information about child mental health daily or weekly [86]. Thus, one could assume that with such a long duration of symptoms, parents had likely already gathered information before app use, which might have resulted in a small effect on the knowledge level in our sample. Future research will have to corroborate this assumption.

### Strengths and Limitations

Evaluating the app’s effectiveness using a RCT design is considered the gold standard for testing the effectiveness of new interventions [106]. However, to date, few RCT intervention studies have been conducted regarding early childhood crying, sleeping, and feeding problems. To the best of our knowledge, this is the first randomized controlled clinical study to target an app-based intervention for families having children with crying, sleeping, and feeding problems as common symptom patterns in early childhood. The newly developed app stands out from other web-based offers because it contains evidence-based strategies; addresses the complexity of co-occurring symptoms of crying, sleeping, and feeding; and combines psychoeducational input with interactive elements. Data collection for effectiveness testing was based on validated measurements. To avoid sequence effects, the questionnaires were administered in permuted order.

However, our results should be interpreted against the background of some limitations of the study. First, it must be mentioned that the study was conducted in a specific clinical setting, and predominantly, mothers of German nationality who had a high level of education and were in a partnership with a sense of social support and self-efficacy participated in the study. Caution is necessary when generalizing our results to other clinical populations. However, these sample characteristics are consistent with other studies on early childhood crying, sleeping, and feeding problems, which also report predominantly academic or higher-educated families in stable partnerships [107,108].

Furthermore, regarding post hoc power, given our sample size, only medium-size effects could be detected with sufficiently high probability (*β*=.82 to .99 for medium effects), whereas power was too low to detect small effect sizes with sufficient probability (*β*=up to .79 for small effects).

Finally, because the study was implemented in a clinical setting, a variation in individual study duration is evident; the measurements were linked to clinic appointments and thus linked to individual variance and deviation because of appointment postponements. However, as pointed out in the Medical Research Council guidelines [63], ensuring very strict standardization might not be appropriate, and a specified degree of adaptation to local settings is preferred.

### Conclusions

In its first practical use in an RCT, a psychoedcuational app for parents of children with crying, sleeping, and feeding problems contributes to parents’ better understanding of their child’s symptoms, has the potential to take the edge off parenting stress, and could therefore also serve as an effective secondary preventive measure. It can be recommended by pediatricians, gynecologists, maternity clinics, or child welfare services as an initial low-threshold information and support service when the first symptoms are developing, or it can be used to bridge waiting times for professional counseling appointments. In the future, the app will be made available free of charge as a low-threshold offer.

## Appendix

Multimedia Appendix 1 CONSORT eHEALTH checklist (V 1.6.1).

Multimedia Appendix 2 Trial registration.

Multimedia Appendix 3 Knowledge test face validity and sensitivity analysis results.

## Abbreviations

CFS: Questionnaire for Crying, Feeding, and Sleeping
CONSORT: Consolidated Standards of Reporting Trials
CONSORT-EHEALTH: Consolidated Standards of Reporting Trials of Electronic and Mobile Health Applications and Online Tele Health
DRKS: German Register of Clinical Studies
EBI: Eltern-Belastungs-Inventar
F-SozU: Social Support Questionnaire
IG: intervention group
ITT: intention-to-treat
PMP-SE: Perceived Maternal Parenting Self-Efficacy Questionnaire
RCT: randomized controlled trial
t1: baseline test
t2: posttest
WCG: waitlist control group
