# Supplementary material for: Effects of a Mobile-Based Intervention for Parents of Children With Crying, Sleeping, and Feeding Problems: Randomized Controlled Trial
Source: JMIR Mhealth Uhealth. 2023 Mar 10;11:e41804. doi: 10.2196/41804 (PMC10039405; doi:10.2196/41804)
Supplement: Multimedia Appendix 2 [file mhealth_v11i1e41804_app2.pdf]

Deutsches Register Klinischer Studien
German Clinical Trials Register

***PLEASE NOTE:** This trial has been registered retrospectively.*

Trial Description

Title

Smartphone-based psychoeducation for parents of children with regulatory problems

Trial Acronym

[---]*

URL of the trial

https://kbo-kinderzentrum-muenchen.de/index.php?id=151&L=3%2Fwp-login.php

Brief Summary in Lay Language

Infant regulatory problems, specifically excessive crying, sleeping and/or feeding problems, are often highly stressful for children and their parents. In many cases, parents feel that they lack self-efficacy and competence when interacting with their child. Additionally, they often have to deal with the perception of being socially isolated. The innovative app „Unser kleiner Schreihals“ provides access to scientifically sound information (by including texts, videos and helpful hints for handling a child with regulatory problems). Furthermore, it contains a diary-function for child symptom documentation, a parent chat forum, tips on relaxation for stressed parents, as well as a list with regional points of contact.

The aim of the study is to put the app „Unser kleiner Schreihals“ to a first practical test. Therefore, we want to investigate whether parents using the app feel less stressed (1), gain more knowledge about regulatory problems (2), perceive themselves as more self-efficient (3), if their child’s symptoms decrease more (4) and if they perceive themselves as less socially isolated (5) than parents who do not use the app.

Therefore, 136 parents of children (age 0 – 24 months) contacting the cry baby outpatient clinic of kbo-Kinderzentrum München for a first consultation are invited to participate in the study. During the waiting time for their appointment (average 3 weeks), one group gets access to the app right away, whereas a comparison group gets access to the app only after their first consultation (= study end). Both groups are examined using questionnaires at two points of measurement (before and after using the app/ waiting time without using the app). Moreover, an evaluation questionnaire assesses participants‘ user behavior as well as user easiness and conception of the app.

After finalization of the study, the app will be provided free of charge and can be used as an additional support, e.g. in addition to professional guidance. Specific education about regulatory problems and treatment options might encourage parents to seek professional support at an early stage.

Brief Summary in Scientific Language

Infant regulatory problems, specifically excessive crying, sleeping and/or feeding problems, are often highly stressful for children and their parents. In many cases, parents feel that they lack self-efficacy and competence when interacting with their child. Additionally, they often have to deal with the perception of being socially isolated.

The innovative app „Unser kleiner Schreihals“ provides a low-threshold access to scientifically sound psychoeducative information (by including texts, videos and helpful hints for handling a child with regulatory problems). Furthermore, it contains a diary-function for child symptom documentation, a parent chat forum, tips on relaxation for stressed parents, as well as a list with regional points of contact.

The aim of the study is to put the app „Unser kleiner Schreihals“ to a first practical test. Therefore, we want to investigate whether parents using the app feel less stressed (1), gain more knowledge about regulatory problems (2), perceive themselves as more self-effective (3), if their child’s symptoms decrease more (4) and if they perceive themselves as less socially isolated (5) than parents who do not use the app.
The efficacy of the app is examined in a monocentre, prospective, randomized-controlled trial (RCT) with a waiting control group design. The project is funded by „Gesund.leben.Bayern“, an initiative by the Bavarian Ministery of Health. Therefore, N = 136 (N = 170 incl. 20% dropouts) parents of children (age 0 – 24 months) contacting the cry baby outpatient clinic of kbo-Kinderzentrum München for a first consultation are recruited. During the waiting time for their appointment (average 3 weeks), the intervention group gets access to the app right away, whereas the waiting control group gets access to the app only after their first consultation appointment (= study end). Both groups are compared regarding parental stress levels, knowledge about regulatory problems, perceived self-efficacy, child symptoms as well as perceived parental social support using questionnaires at two points of measurement (t1 = pre; t2 = post). Moreover, an evaluation questionnaire assesses participants‘ user behavior as well as user easiness and conception of the app.

After finalization of the study, the app will be provided free of charge and can be used as an additional support, e.g. in addition to professional guidance. Specific education about regulatory problems and treatment options might encourage parents to seek professional support at an early stage.

Do you plan to share individual participant data with other researchers?

No

Description IPD sharing plan

[---]*

Organizational Data

- DRKS-ID: **DRKS00019001**
- Date of Registration in DRKS: **2020/01/28**
- Date of Registration in Partner Registry or other Primary Registry: **[---]***
- Investigator Sponsored/Initiated Trial (IST/IIT): **yes**
- Ethics Approval/Approval of the Ethics Committee: **Approved**
- (leading) Ethics Committee Nr.: **56/18 S , Ethik-Kommission der Fakultät für Medizin der Technischen Universität München**

Secondary IDs

- Universal Trial Number (UTN): **U1111-1241-7567**

Health condition or Problem studied

- Other: **DC:0-5: excessive crying disorder, sleep onset disorder, night waking disorder, undereating disorder, other sleep, eating and excessive crying disrder of infancy/ early childhood**

Interventions/Observational Groups

- Arm 1: **Intervention group:**

  The intervention group gets exclusive and unlimited access to the app "Unser kleiner Schreihals" during the waiting period until the first consultation in the cry baby outpatient clinic of kbo-Kinderzentrum (average 3 weeks).

- Arm 2: **Waiting control group: no app-usage**

  The waiting control group does not get immediate access to the app during their waiting time until the first consultation in the cry baby outpatient clinic of kbo-Kinderzentrum. Unlimited access is provided after the first consultation appointment (= study end).

Characteristics

- Study Type: **Interventional**
- Study Type Non-Interventional: **[---]***
- Allocation: **Randomized controlled trial**
- Blinding: **[---]***
- Who is blinded: **investigator/therapist**
- Control: **Control group receives no treatment**

Study Type: **Interventional**

Study Type Non-Interventional: **[---]***

Allocation: **Randomized controlled trial**

Blinding: **[---]***

Who is blinded: **investigator/therapist**

Control: **Control group receives no treatment**

- Purpose: **Prevention**
- Assignment: **Parallel**
- Phase: **N/A**
- Off-label use (Zulassungsüberschreitende Anwendung eines Arzneimittels): **N/A**

Primary Outcome

post-treatment (t2); compared to pre-treatment (t1) and to waiting control group:

Parental stress/burden: Score in questionnaire "Eltern-Belastungs-Inventar" (Tröster, 2010; German version of "Parenting Stress Index (PSI)", Abidin, 1983, 2012)

Secondary Outcome

post-treatment (t2); compared to pre-treatment (t1) and to waiting control group:

- Parental knowledge about regulatory problems: Score in questionnaire "Multiple-Choice-Test über Schreien, Schlafen, Füttern" (self-developed instrument using face-validity)

- Perceived parental self-efficacy: Score in questionnaire "The Perceived Maternal Parenting Self-Efficacy Questionnaire (PMP S-E)" (Barnes & Adamson-Macedo, 2007; version professionally translated into German)

- Child symptoms (duration/severity/kind): Score in questionnaire „Schreien, Füttern, Schlafen (SFS)“ (Groß, Reck, Thiel-Bonney, & Cierpka, 2013)

- Perceived parental social support: Score in questionnaire "Fragebogen zur Sozialen Unterstützung (F-SozU)" (Fydrich, Sommer & Brähler, 2007; short form containing 22 items)

Sample description/ covariates:

- General information: Questionnaire containing demographic information and cause for contacting crying ambulance (t1)

- App-Evaluation: Evaluation questionnaire of app-usage (t2)

- Clinical diagnosis according to DC:0-5 (Zero to Three, 2016) (after first consultation appointment)

Countries of recruitment

- DE **Germany**

Locations of Recruitment

- Medical Center **kbo-Kinderzentrum München; Lehrstuhl für Sozialpädiatrie der Technischen Universität München, München**

Recruitment

- Planned/Actual: **Actual**
- (Anticipated or Actual) Date of First Enrollment: **2019/02/21**
- Target Sample Size: **136**
- Monocenter/Multicenter trial: **Monocenter trial**
- National/International: **National**

Inclusion Criteria

- Gender: **Both, male and female**
- Minimum Age: **0 Months**
- Maximum Age: **24 Months**

Additional Inclusion Criteria

contact in the run-up to a first consultation in the cry baby outpatient clinic of kbo-Kinderzentrum.

regulatory problems (crying, sleeping and/or feeding)

Exclusion criteria

child older than 24 months;

no consent for study participation

Addresses

- **Primary Sponsor**

Lehrstuhl für Sozialpädiatrie der Technischen Universität/ Klinikum rechts der Isar München
Ismaninger Str. 22
81675 München
Germany

Telephone: **[---]***
Fax: **[---]***
E-mail: **[---]***
URL: **https:/kbo-kinderzentrum-muenchen.de/index.php?id=98**

- **Contact for Scientific Queries**

Lehrstuhl für Sozialpädiatrie der Technischen Universität München
Ms. Dr. phil. Anna Friedmann
Heiglhofstr. 65
81377 München
Germany

Telephone: **0049 89 71009 392**
Fax: **[---]***
E-mail: **anna.friedmann at kbo.de**
URL: **https://kbo-kinderzentrum-muenchen.de/**

- **Contact for Public Queries**

Lehrstuhl für Sozialpädiatrie der Technischen Universität
Ms. M. Sc. Michaela Augustin
Heiglhofstr. 65
81377 München
Germany

Telephone: **0049 89 71009 - 392**
Fax: **[---]***
E-mail: **michaela.augustin at kbo.de**
URL: **https://kbo-kinderzentrum-muenchen.de/**

Deutsches Register Klinischer Studien
German Clinical Trials Register

- **Collaborator, Other Address**

  The University of Warwick Department of Psychology – Lifespan Health and Wellbeing Group and Division of Health Sciences, Warwick Medical School
  Mr. Prof. Dr. Dieter Wolke
  CV4 7AL Coventry
  United Kingdom

  Telephone: **[---]***
  Fax: **[---]***
  E-mail: **D.Wolke at warwick.ac.uk**
  URL: **https://warwick.ac.uk/fac/sci/psych/**

Sources of Monetary or Material Support

- **Public funding institutions financed by tax money/Government funding body (German Research Foundation (DFG), Federal Ministry of Education and Research (BMBF), etc.)**

  Bayerisches Staatsministerium für Gesundheit und Pflege
  Haidenauplatz 1
  81677 München
  Germany

  Telephone: **[---]***
  Fax: **[---]***
  E-mail: **[---]***
  URL: **[---]***

Status

- Recruitment Status: **Recruiting complete, follow-up complete**
- Reason, if "Recruitment stopped after recruiting started" or "Recruiting withdrawn before recruting started": **[---]***
- Reason, if Reason for Recruiting Stop "Other": **[---]***
- Study Closing (LPLV): **2022/01/13**
- Number of Participants in Germany after Recruiting complete: **136**
- Total Number of Participants (all Sites worldwide) after Recruiting complete: **136**

Trial Publications, Results and other documents
