# Supplementary material for: Effects of a Mobile-Based Intervention for Parents of Children With Crying, Sleeping, and Feeding Problems: Randomized Controlled Trial
Source: JMIR Mhealth Uhealth. 2023 Mar 10;11:e41804. doi: 10.2196/41804 (PMC10039405; doi:10.2196/41804)
Supplement: Multimedia Appendix 3 [file mhealth_v11i1e41804_app3.docx]

**Multimedia Appendix 3. Knowledge test face validity and sensitivity analysis results.**

This is a Multimedia Appendix to a full manuscript published in the J Med Internet Res. For full copyright and citation information see http://dx.doi.org/10.2196/jmir. 41804.

| **Table S1**. Knowledge Test - Face validity | | |
| --- | --- | --- |
| Knowledge test item^a^ | Eligibility rating^b^ | |
|  | Md^b^ | M (SD)^b^ |
| 1. “Infant/toddler crying can have several causes. Which of the statement/s is/are correct?” | 5 | 4,42 (0.65) |
| 1. “It is natural for infants to cry (e.g. due to tummy ache). However, at a certain age, the crying frequency decreases in most infants. When does this occur?” | 4 | 4.11 (0.83) |
| 1. “Some infants/toddlers cry persistently and for no apparent reason (“excessive crying”). There are three scientifically based criteria that are often used to evaluate excessive crying (so-called Wessel criteria). What are the Wessel criteria?” | 4 | 3.53 (1.17) |
| 1. “Some infants/toddlers continue to cry even after their parents have tried everything to calm them down. Which of the following is typical in infants/toddlers who cry excessively?” | 4 | 4.24 (0.73) |
| 1. “You have probably already heard a lot about crying in infants/toddlers. However, not all of it might be true. Which of the statement/s is/are false?" | 4 | 3.93 (0.92) |
| 1. “How can you help your infant/toddler when he/she cries a lot?” | 4 | 4.30 (0.79) |
| 1. “If your baby/toddler cries a lot, you may often be able to come up with helpful solutions on your own. However, there may be times when you can no longer cope on your own. When should you definitely seek professional help?” | 5 | 4.41 (0.73) |
| 1. “Which of the statements about the sleep of infants/toddlers is/are correct?” | 4 | 4.14 (0.71) |
| 1. “Falling asleep is difficult for many infants/toddlers. From the age of 8 months, persistent sleep onset problems may occur. What are the characteristics of persistent sleep onset problems?" | 4 | 4.20 (0.75) |
| 1. “Many children have difficulties maintaining sleep. Which of the statements is/are correct regarding persistent problems maintaining sleep (from the age of 8 months)?” | 4 | 4.06 (0.79) |
| 1. “The sleep rhythm of infants/toddlers differs from that of adults. Which of the statement/s about infant’s/toddler’s night sleep is/are correct?” | 4 | 4.17 (0.73) |
| 1. “How can you help your infant/toddler if he/she is having sleeping problems?" | 4 | 4.38 (0.71) |
| 1. “What should you be aware of when your infant/toddler is showing difficulties sleeping?” | 4 | 4.30 (0.74) |
| 1. “Infant/toddler eating habits differ from those of adults. Which of the statements about the development of eating behavior of infants/toddlers is/are correct?” | 4 | 4.05 (0.77) |
| 1. “Feeding problems are often transient and symptoms decrease after a short time. During which situations/time periods are feeding problems likely to occur for the first time or to become more severe?” | 4 | 3.98 (0.81) |
| 1. “Sometimes feeding problems persist. Which of the statements about persistent feeding problems is/are true?" | 4 | 3.94 (0.90) |
| 1. “How can you help if your infant/toddler is having problems with feeding/eating problems?" | 4 | 4.27 (0.77) |
| 1. “After birth, feeding is initially all about breastfeeding or bottle-feeding. Which of the following statements is/are correct?” | 4 | 3.89 (0.97) |
| ^a^All knowledge test items were followed by multiple choice answers with one or more possible correct answers.  ^b^ Knowledge test items were rated by parents for their eligibility using the item “The knowledge test has been developed by our study team. We are therefore interested in how suitable you consider the questions to be in order to assess your knowledge level in the areas of crying, sleeping, and feeding” based on 5-points-Likert scales (1= not suitable at all, 2 = mostly not suitable 3 = partly suitable, 4 = mostly suitable, 5 = very suitable) | | |

| **Table S2.** Analysis of covariance (ANCOVA) results for completer sample. | | | | | | | |
| --- | --- | --- | --- | --- | --- | --- | --- |
|  | | | | | | | |
| Variable | | *n* | *F (df)* | *P* value | | *d* | *95% CI* |
| EBI^c^ parental subscale | | 112 | 5.632 (1, 108) | .01^a^ | | -0.37 | -0.69 to -0.06 |
|  | Attachment | 112 | 0.001 (1, 109) | .97 | -0.06 | | -0.43 to 0.31 |
|  | Isolation | 112 | 8.694 (1, 109) | .004^b^ | -0.48 | | -0.86 to -0.10 |
|  | Parental competence | 112 | 0.590 (1, 109) | .44 | -0.18 | | -0.55 to 0.20 |
|  | Depression | 112 | 0.193 (1, 109) | .66 | -0.01 | | -0.38 to 0.36 |
|  | Health | 112 | 3.729 (1, 109) | .06 | -0.34 | | -0.71 to 0.04 |
|  | Role restriction | 112 | 4.307 (1, 109) | .04^b^ | -0.34 | | -0.71 to 0.04 |
|  | Spouse-related stress | 112 | 6.871 (1, 109) | .01^b^ | -0.52 | | -0.89 to -014 |
| Knowledge test | | 109 | 29.273 (1, 106) | <.001^b^ | | 0.54 | 0.15 to 0.92 |
| PMP-SE^d^ | | 105 | 1.434 (1, 102) | .23 | | 0.21 | -0.18 to 0.59 |
| F-SozU^e^ | | 113 | 1.633 1, 110) | .20 | | 0.11 | -0.06 to 0.28 |
| CFS^f^ | | 56 | 1.082 (1, 52) | .30 | | 0.14 | -0.39 to 0.66 |
| ^a^1-tailed test based on the t-distribution.  ^b^2-tailed test (F-test).  ^c^EBI: Eltern-Belastungs-Inventar.  ^d^PMP-SE: Perceived Maternal Parenting Self-Efficacy Questionnaire.  ^e^F-SozU: Social Support Questionnaire.  ^f^CFS: Questionnaire for Crying, Feeding and Sleeping. | | | | | | | |

| **Table S3.** Analysis of covariance (ANCOVA) results for per protocol sample. | | | | | |
| --- | --- | --- | --- | --- | --- |
|  | | | | | |
| Variable | | *F (df^a^)* | *P* value | *d* | *95% CI* |
| EBI^d^ parental subscale | | 3.24 (1, 6089.04) | .04^b^ | -.21 | -0.51 to 0.09 |
|  | Attachment | 0.22 (1, 6580.01) | .64 | .04 | -0.30 to 0.38 |
|  | Isolation | 4.83 (1, 3123.05 | .03^c^ | -.34 | -0.69 to -0.00 |
|  | Parental competence | 0.30 (1, 4930.84) | .58 | -.04 | -0.38 to 0.30 |
|  | Depression | 0.07 (1, 21299.95) | .80 | .11 | -0.23 to 0.46 |
|  | Health | 2.47 (1, 3104.54) | .12 | -.23 | -0.58 to 0.11 |
|  | Role restriction | 2.03 (1, 7448.44) | .14 | -.21 | -0.56 to 0.13 |
|  | Spouse-related stress | 4.25 (1, 2662.69) | .04^c^ | -.39 | -0.74 to – 0.05 |
| Knowledge test | | 26.79 (1, 1672.71) | <.001^c^ | .53 | 0.19 to 0.89 |
| PMP-SE^e^ | | 0.45 (1, 22941) | .45 | .02 | -0.32 to 0.36 |
| F-SozU^f^ | | 0.89 (1, 27897.47) | .35 | .08 | -0.08 to 0.23 |
| CFS^g^ | | 0.57 (1, 550.87) | .45 | .19 | -0.15 to 0.54 |
| ^a^ Approximated *df* based on multivariate imputation  ^b^1-tailed test based on the t-distribution.  ^c^2-tailed test (F-test).  ^d^EBI = Eltern-Belastungs-Inventar.  ^e^PMP-SE: Perceived Maternal Parenting Self-Efficacy Questionnaire.  ^f^F-SozU: Social Support Questionnaire.  ^g^CFS: Questionnaire for Crying, Feeding and Sleeping. | | | | | |
